# Supplementary material for: Reconciling Mining with the Conservation of Cave Biodiversity: A Quantitative Baseline to Help Establish Conservation Priorities
Source: PLoS One. 2016 Dec 20;11(12):e0168348. doi: 10.1371/journal.pone.0168348 (PMC5173368; doi:10.1371/journal.pone.0168348)
Supplement: S1 Dataset — (ZIP) [file pone.0168348.s002.zip › Taxa/Serra Norte/SN_2013/N4E/Lista N4E-53.pdf]

CAVIDADE N4E-0053

Classe	Ordem	Fam/Outros	Gên/Outros	Espécie	S-U	U-S	Zon.
Arachnida	Amblypygi	Phrynidae	*Heterophrynus*	*longicornis*	1		E
Arachnida	Araneae	Corinnidae		jovem		1	E
Arachnida	Araneae	Ctenidae	*Nothroctenus*	*marshi*	2	1	E
Arachnida	Araneae	Filistatidae		sp.1	X	X	E
Arachnida	Araneae	Ochyroceratidae	*Ochyrocera*	sp.3	X	X	E
Arachnida	Araneae	Pholcidae	*Leptopholcus*	sp.1	X	X	E
Arachnida	Araneae	Pholcidae	*Mesabolivar*	aff. *cambridgei*	X		E
Arachnida	Araneae	Pholcidae	*Mesabolivar*	sp.1	X	X	E
Arachnida	Araneae	Pholcidae	Ninetinae	sp.1	X	X	E
Arachnida	Araneae	Scytodidae	*Scytodes*	jovem	1		E
Arachnida	Araneae	Segestriidae	*Ariadna*	sp.1	X		E
Arachnida	Opiliones	Stygnidae	*Protimesius*	*laevis*	1		E
Arachnida	Pseudoscorpiones	Chernetidae	*Spelaeochernes*	sp.1	X	X	E
Entognatha	Collembola	Paronellidae		sp.4		X	E
Insecta	Coleoptera			jovem	X		E
Insecta	Diptera	Psychodidae	Phlebotominae	sp.		X	E
Insecta	Diptera	Tipulidae	Tipulinae	sp.	X	X	E
Insecta	Hymenoptera	Formicidae	*Camponotus*	sp.2	X		E
Insecta	Hymenoptera	Formicidae	*Pachycondyla*	sp.2	X	X	E
Insecta	Isoptera			sp.	X		E
Insecta	Lepidoptera	Galacticidae		sp.		X	E
Insecta	Lepidoptera	Noctuoidea		sp.5		1	E
Insecta	Lepidoptera	Roeslerstammiidae		sp.		X	E
Insecta	Orthoptera	Phalangopsidae	*Paraclodes*	sp.	5		E
Insecta	Orthoptera	Phalangopsidae	*Phalangopsis*	sp.	1		E
Insecta	Thysanura	Nicoletiidae		jovem		X	E
